# Analysis of Influencing Factors on the Gas Separation Performance of Carbon Molecular Sieve Membrane Using Machine Learning Technique

**DOI:** 10.3390/membranes12010100

**Published:** 2022-01-17

**Authors:** Yanqiu Pan, Liu He, Yisu Ren, Wei Wang, Tonghua Wang

**Affiliations:** 1 School of Chemical Engineering, Dalian University of Technology, Dalian 116024, China; yqpan@dlut.edu.cn (Y.P.); liuhe1992@hotmail.com (L.H.); wangth@dlut.edu.cn (T.W.); 2 Jihua Laboratory, Foshan 528000, China; 3 Faculty of Science, The University of Melbourne, Melbourne, VIC 3010, Australia; szrenyisu@hotmail.com

**Keywords:** carbon molecular sieve membrane, gas separation, machine learning, support vector regression

## Abstract

Gas separation performance of the carbon molecular sieve (CMS) membrane is influenced by multiple factors including the microstructural characteristics of carbon and gas properties. In this work, the support vector regression (SVR) method as a machine learning technique was applied to the correlation between the gas separation performance, the multiple membrane structure, and gas characteristic factors of the self-manufactured CMS membrane. A simple quantitative index based on the Robeson’s upper bound line, which indicated the gas permeability and selectivity simultaneously, was proposed to measure the gas separation performance of CMS membrane. Based on the calculation results, the inferred key factors affecting the gas permeability of CMS membrane were the fractional free volume (FFV) of the precursor, the average interlayer spacing of graphite-like carbon sheet, and the final carbonization temperature. Moreover, the most influential factors for the gas separation performance were supposed to be the two structural factors of precursor influencing the porosity of CMS membrane, the carbon residue and the FFV, and the ratio of the gas kinetic diameters. The results would be helpful to the structural optimization and the separation performance improvement of CMS membrane.

## 1. Introduction

Membrane-based gas separation technology has been widely concerned because of its high separation efficiency, environmental friendship, and easy operation [1,2]. In order to broaden the application of the technology, it is important to develop novel membrane materials with excellent gas separation performance [3]. Carbon molecular sieve (CMS) membrane is a carbon-based membrane fabricated from the pyrolysis of polymeric precursor film [4,5]. As a novel membrane for gas separation with a broad development prospect, CMS membrane has the advantages of excellent gas permeability and selectivity, high thermal and chemical stability, and anti-plasticization. The microstructure of CMS membranes varies largely with the choice of precursors and preparation processes, affecting the separation performance [5,6,7,8]. Exploring the influence of factors, such as the microstructural characteristics and gas properties, on the gas separation performance, or the so-called permselectivity, would be helpful to the adjustment of the microstructure and the optimization to the membrane preparation. However, gas permeability of CMS membrane is hard to correlate functionally with the plural factors which are not directly interrelated [9,10,11]. The machine learning technique, which is the core of artificial intelligence, makes it possible to realize the correlation [12,13].

Machine learning is a computational technology to quantitatively predict the relationship between the multiple conditions and the target based on given samples [13,14]. In the fields of biomedicine, chemistry, materials, and the environment, the machine learning technique has been successfully applied to the analysis of the relationship between the characteristics and the performances of materials [15,16,17,18,19]. Specific to membrane-based gas separation, machine learning has been applied to the performance prediction and structural optimization of polymer membranes, zeolite membranes, metal-organic framework membranes, and composite membranes [20,21,22,23,24,25,26]. For CMS membrane, Behnia et al. [27] predicted the gas permeability and selectivity through statistical analysis and modeling based on five influential factors, including the type of precursor, blend composition of precursors, final pyrolysis temperature, vacuum pressure during pyrolysis, and operating pressure. The authors concluded that every factor has a significant impact on the gas permeability, especially the final pyrolysis temperature. Based on the actual measurement data of CMS membrane, the machine learning technique is feasible to discuss the influences of structural characteristics, gas properties, and other factors on the gas permeation and separation of CMS membrane.

Several methods based on machine learning techniques were used to analyze the influence of plural factors on specific performance of material based on the obtained data in recent years, such as multiple linear regression, partial least-square, multiple discrimination vector, decision tree, and support vector regression [28,29,30,31,32,33]. The support vector regression (SVR) method could seek the best compromise between the complexity of the model and the learning ability to obtain the best promotion ability from the limited sample. The SVR method has the following advantages [13,14,28]: (1) for small samples, it could achieve the optimal solution through the existing information; (2) it could get the global optimal solution in theory; and (3) the complexity of the SVR method is independent of the sample dimension. The SVR method is anticipated to effectively analyze the influence of the factors, including structural characteristics and gas properties on the gas separation performance of the CMS membrane based on limited actual characterization results and experimental data. 

In this work, the SVR method was applied to the analysis of the multiple factors influencing the gas separation performance of CMS membranes. The data for analysis were collected from the authors’ previous work [34,35,36,37,38,39,40,41,42,43]. The monomer structure formulas of the polymeric precursors for preparing CMS membranes are shown in Figure S1. The gas permeability and separation performance of CMS membrane were predicted through the factors, and the key influencing factors were determined. The method and results in this work may provide a new insight into the structure-performance relationship of CMS membrane for gas separation.

## 2. Computational Methods

### 2.1. Data Collection

Since the existing characterization and measurement data were limited in the category, some structural factors of CMS membranes were substituted by the related ones of precursors. Most permeation coefficients in the previous works were measured at 30 °C and 100 kPa (375 results selected as the sample in this work), and only a small amount of data (24 results) was obtained under other conditions [34,35,36,37,38,39,40,41,42,43]. The influence of structural factors and operating conditions was analyzed in the follow-up work.

Table 1 shows the influencing factors to be discussed as the independent variables based on the structural characterization and gas permeability [34,35,36,37,38,39,40,41,42,43], including the precursor characteristic, carbonization condition, carbon microstructure, and molecular property of permeated gases. Among the characteristics of polymeric precursors, the fractional free volume (FFV) and the carbon residue influencing the porosity of CMS membrane were selected as the influential factors [44,45,46], as well as the fraction of sp2 carbon and aromatic carbon affecting the arrangement of carbon molecules [47,48]. The pyrolysis temperature, which is particularly applicable to the structural adjustment of CMS membrane, was chosen as an influencing factor [49]. The carbon microstructural factors contained the average interlayer spacing, length, and thickness of the carbon microcrystal consisting CMS membrane [6,9]. In addition, the gas molecular kinetic diameter related to the resistance, the gas molecular mass affecting the free diffusion, and the gas–carbon interaction potential influencing the gas sorption in CMS membrane were taken into consideration [50,51,52]. The calculation methods of some factors are depicted in S.2–S.4 of the Supplementary Materials.

**Figure 1:**
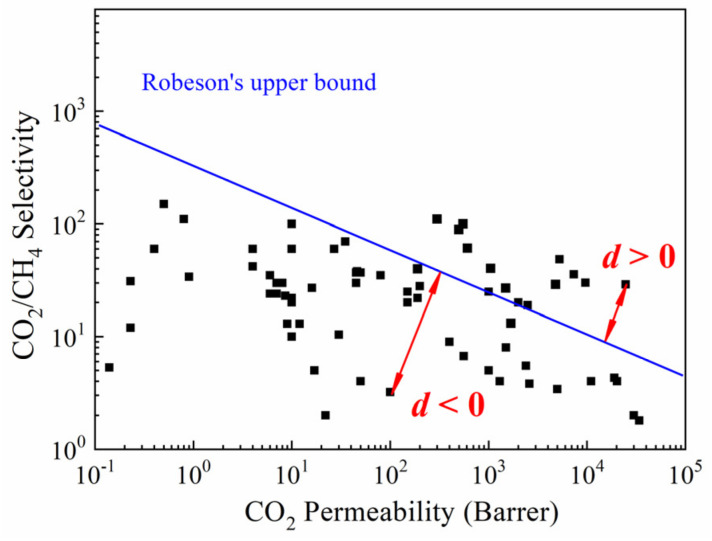
Permselectivity values (characteristic distance *d*) of the CO_2_/CH_4_ gas pair based on Robeson’s upper bound [54].

As the performance indicator (dependent variable) of CMS membranes, besides the permeability of gases (CO_2_, CH_4_, N_2_, O_2_, and H_2_), a quantitative index, named characteristic distance (*d*), was proposed in this work. Figure 1 shows the concept of *d*, which was the vertical distance of a black legend away from the Robeson’s upper bound line of a specific gas pair [53,54]. When the legend was above the upper bound line, the *d* was a positive value; otherwise, the *d* was negative. The *d*, as a quantitative index, could measure the gas separation performance of CMS membranes, including permeability and selectivity simultaneously.

The formula of Robeson upper bound line is [54]:

(1) lgP=lgk+nlgα

where *P* (Barrer, 1 Barrer = 10^−10^ cm^3^(STP)·cm·cm^−2^·s^−1^·cmHg^−1^) is the permeability coefficient of fast gas; *α* is the selectivity coefficient; and *n* and lg*k* are the slope and intercept of the upper bound line, respectively. In order to make a consistent evaluation standard to different gas pairs, the *n* and lg*k*, as shown in Tables S2 and S5 in the Robeson upper bound lines [54], were selected for all gas pairs in the sample.

**Figure 2:**
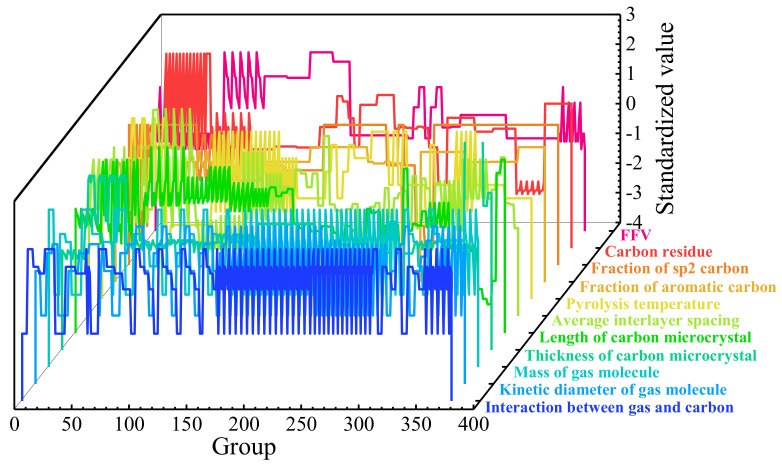
Standardized values of the independent variables.

In order to ensure the comparability between different variables and the reasonability of the regression results, the variable *x* should be transferred to *X* with the mean value of 0 and the standard deviation of 1:

(2) $$X=\frac{x-\mu }{\sigma }$$

where *μ* and *σ* are the mean value and the standard deviation of the variable *x*, respectively. The standardized results of the variables are shown in Figure 2, which were used to show the relevance of variables qualitatively and illustrate the independence of them. Obviously, variations of different independent variables with the group number were inconsistent and did not follow a specific variation trend. Thus, it could be considered that the collected data were suitable for calculation.

### 2.2. SVR Theorem

**Figure 3:**
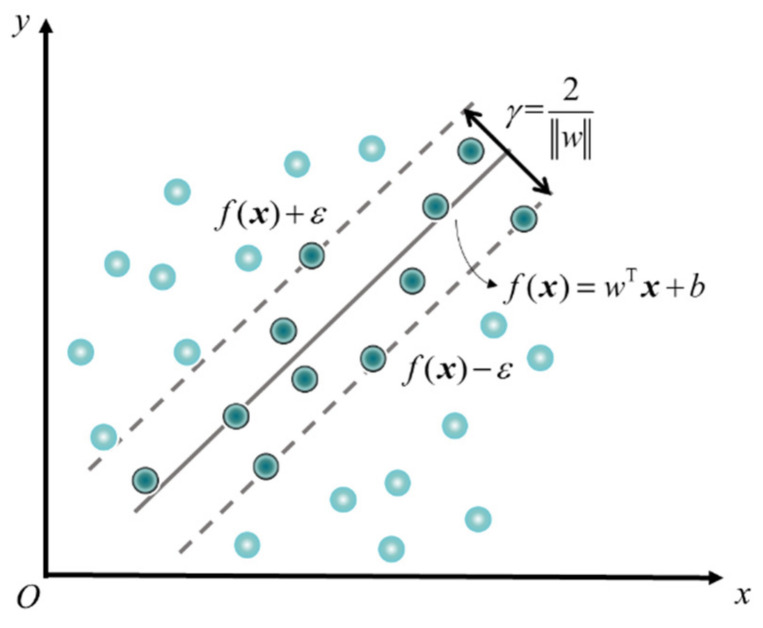
Diagram of SVR theorem.

Figure 3 indicates the basic idea of the SVR method. For independent variables ***x*** and dependent variable *y*, the loss is calculated only if the difference between *y* and the results predicted from the independent variable *f*(***x***) are greater than *ε* [13]. When |*f*(***x***) − *y*| < *ε*, the value of |*f*(***x***) − *y*| is treated as zero; otherwise, the value is |*f*(***x***) − *y*| − *ε*. It is equivalent to building a 2*ε* wide belt centered on *f*(***x***) and the prediction is correct when the sample falls into the belt. The *f*(***x***) is called hyperplane, whose geometry is related to the dimension of ***x***. However, the hyperplane may not be constructed in practice due to the characteristics of the sample. Under such circumstances, the samples could be mapped from the original sample space to a higher dimensional feature space in order to realize the linear correlation. The *γ* is called breadth, which affects the range of action and thereby affects the generalization performance.

In the sample space, the hyperplane could be described by:

(3) $$f(x)=w^{T}x+b$$

where *w* is the vector of weight (*w*^T^ is the transpose of *w*), and *b* is the bias term. When the sample needs mapping in order to construct a hyperplane, the original independent ***x*** would map into the eigenvector *Φ*(***x***) and the hyperplane function becomes [13,28]:

(4) $$f(x)=w^{T}\Phi (x)+b$$

The purpose of the SVR is to find the hyperplane with the largest breadth (*γ* in Figure 3), which is equivalent to find the *w* satisfying the objective function min‖*w*‖^2^/2 subject to *y_i_*·(*w*^T^*x*_i_ + *b*) ≥ 1. In practice, however, the hyperplane is difficult to appear in a linear form. There are two ways to make the sample linear regression: adding a penalty coefficient to the objective function and mapping the samples to the higher dimensional space. Then, the objective function of SVR hyperplane becomes:

(5) $$\underset{w,b,\xi_{i},\hat{\xi_{i}}}{\min}\left[\frac{{\left\|w\right\|}^{2}}{2}+C\sum_{i=1}^{m}\left(\xi_{i}+\hat{\xi_{i}}\right)\right]$$

$$s.t.\;\left\{\begin{array}{l} f(x_{i})-y_{i}\leq \varepsilon +\xi_{i}\\ y_{i}-f(x_{i})\leq \varepsilon +\hat{\xi_{i}}\\ \xi_{i}\geq 0,\;\hat{\xi_{i}}\geq 0 \end{array}\right.$$

where *C* is the penalty coefficient, which is the tolerance for the error. The *C* should be neither too large nor too small in order to prevent over-fit and under-fit. *ξ_i_* and $\hat{\xi_{i}}$ are called slack variables, which indicate that the relaxation degree on both sides of the space might be different.

Combined with the rationale of SVR as shown in Figure 3, the solution of the SVR model would be achieved by constructing the Lagrange equation and solving its partial derivative [13]. The Equation (4) could be written in the explicit form:

(6) $$f(x)=\sum_{i=1}^{m}({\hat{\alpha }}_{i}-\alpha_{i})\Phi {\left(x_{i}\right)}^{T}\Phi (x)+b$$

where *α_i_* and ${\hat{\alpha }}_{i}$ are the Lagrange multipliers. The *w* would, therefore, be solved and used in the comparison of each influencing factor.

In practice, the kernel function *κ*(***x****_i_*, ***x****_j_*), which is a symmetric and positive definite in the sample space, is constructed to replace the inner product of the mapping eigenvectors:

(7) $$\kappa (x_{i},x_{j})=\Phi {\left(x_{i}\right)}^{T}\Phi (x_{j})$$

The kernel function could deal with feature space of arbitrary dimensionality, and the functional form of *Φ*(***x****_i_*), which is hidden in the kernel function, is unnecessary to calculate explicitly [28,55]. Selecting the kernel function would be helpful to correctly construct the hyperplane in feature space. In this work, the SVR models with linear function, polynomial function, radial basis function (RBF), and Sigmoid function in S.6 (Equations (S13)–(S16)) were built as the kernel functions, and the best model was chosen to analyze the influences of the multiple factors on the gas separation performance of CMS membranes.

## 3. Analyzing Process of Influencing Factors

**Figure 4:**
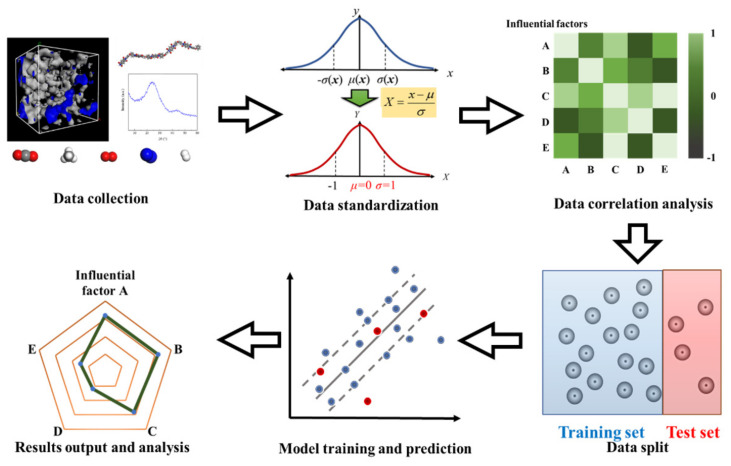
The processing route of influencing factor analysis based on SVR method.

Figure 4 depicts the analyzing process of the multiple influencing factors on gas permeation and separation performance of CMS membrane based on the SVR method. First, the obtained data were collected from the references [34,35,36,37,38,39,40,41,42,43]. The values of some factors, such as FFV, carbon structural parameters, and gas–carbon interaction, should be calculated through S.2–S.4 of the Supplementary Materials. Next, the data of each factor were standardized according to Section 2.1, in order to ensure the comparability between any two independent variables. Then, the correlation coefficients were calculated and the independent variables were eliminated with high correlation. After data elimination, the remaining processed data were randomly divided into a training set (the blue dots) and a test set (the red dots) in the proportion of 4:1 [26,29]. During the calculation, the data of the training set were used to train the model, and the test set was calculated by the trained model to test the model’s reliability. Finally, the important factors affecting the performance were determined and finally analyzed based on the average weight results of 100 times calculation. The analyzing process on the basis of the SVR method was realized using Python script.

## 4. Results and Discussion

### 4.1. Correlation Analysis on the Independent Variables

If the two variables with strong correlation to each other in the sample are simultaneously taken into account in the analysis, the quality of the model may be negatively affected and the regression result would become unreasonable [28]. Before the regression, the correlation coefficient *R*s (Equation (8), where the *Cov*(*x_i_*, *x_j_*) is the covariance of the independent variables *x_i_* and *x_j_*, *σ*^2^(*x_i_*) and *σ*^2^(*x_j_*) and their variations) were calculated, and one of the variables with large correlation coefficient was removed. The value of the correlation coefficient, which assessed whether to remove the variable, was between 0.8 and 0.9 in the references [28,29,56,57,58]. In this work, the value was selected as 0.8, which is also the criterion for judging whether two variables are highly correlated [59].

(8) $$R(x_{i},x_{j})=\frac{Cov(x_{i},x_{j})}{\sqrt{\sigma^{2}(x_{i})\sigma^{2}(x_{j})}}$$

**Figure 5:**
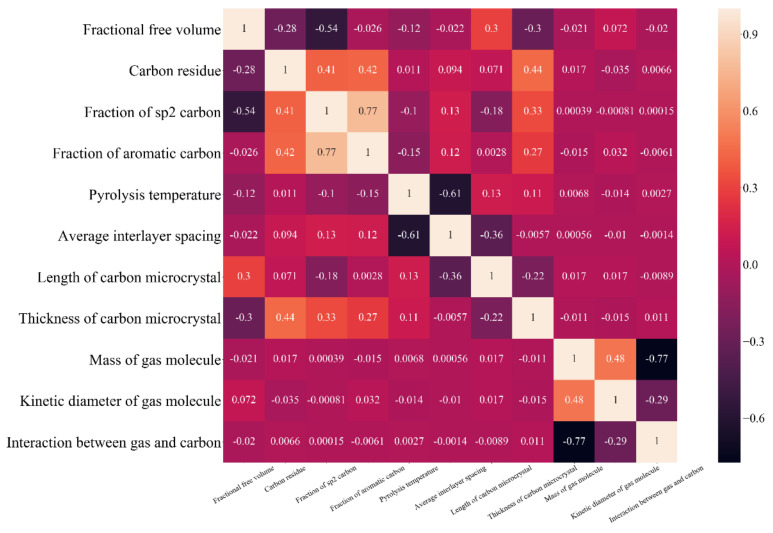
Heatmap of the correlation coefficients between independent variations.

Figure 5 shows the heatmap depicting the calculation results of the correlation coefficients between every two independent variables in the sample data. All the correlation coefficients were less than 0.8. Therefore, no variable was deleted in the processes of model establishing, training, and predicting.

### 4.2. Model Reliability and Parameter Optimization

In order to validate the reliability of the SVR method in the correlation of the influencing factors and gas permeability of CMS membranes, the SVR method with four kinds of kernel function and three classical multiple linear regression (MLR) methods (simple linear regression, Ridge regression, and Lasso regression) were, respectively, used for the regression between the influential factors and the gas permeability of CMS membranes. Three statistical indicators between the calculation results and the experimental data, including the determination coefficient (*R*^2^, Equation (9)), root mean square error (*RMSE*, Equation (10)), and mean absolute error (*MAE*, Equation (11)), were calculated in order to compare the regression effect of each method.

(9) $$R^{2}=1-\frac{\sum_{i=1}^{m}{\left[y_{i}-f(x_{i})\right]}^{2}}{\sum_{i=1}^{m}{\left(y_{i}-\bar{y}\right)}^{2}}$$

(10) $$RMSE=\sqrt{\frac{1}{m}\sum_{i=1}^{m}{\left[y_{i}-f(x_{i})\right]}^{2}}$$

(11) $$MAE=\frac{1}{m}\sum_{i=1}^{m}\left|y_{i}-f(x_{i})\right|$$

where *y_i_* and *f*(*x_i_*) are, respectively, the actual dependent variable and the calculated result based on the independent variable of the *i*th sample; $\overset{—}{y}$ is the mean value of the actual dependent variable; and *m* is the number of samples. The *R*^2^, *RMSE,* and *MAE* of the gas permeabilities between the experimental data and the predicted values by SVR and MLR methods were calculated, and the accuracies of these methods were determined by the calculated results.

Table 2 and Table 3 list the statistical indicators calculated by the SVR method and the MLR method, respectively, based on the experimental data from the authors’ previous work [34,35,36,37,38,39,40,41,42,43]. The calculated indicators of the SVR method, except for the one of the models with the sigmoid kernel, exhibited larger *R*^2^, smaller *RMSE*, and smaller *MAE* than the ones of the MLR method. This indicates that the SVR method with global optimality may correlate the influencing factors and the permeability of the CMS membrane more accurately. In addition, the models with the RBF kernel and the polynomial kernel showed similar regression effects on the influencing factors and the gas permeability of the CMS membrane, but the models with the linear kernel and the sigmoid kernel could not correlate the influencing factors and the performances well. The model with the RBF kernel, which is less complex and could realize non-linear mapping, is slightly better than the one with the polynomial kernel and is more suitable for the regression in this work.

In order to improve the regression performance of the SVR models with the RBF kernel and the polynomial kernel, the parameters of the models needed to be modified. As for the model with the RBF kernel, the penalty coefficient (*C*) in Equation (5) and the breadth (*γ*) in Figure 3 were optimized. The polynomial degree of the polynomial kernel function also needed to be adjusted. The variations of statistical indicators with the model parameters are shown in Figure S3 and Figure S7. Aiming at larger *R*^2^ while avoiding both over-fit and under-fit, *C* = 5 and *γ* = 0.3 were finally selected as the parameters of the SVR model with the RBF kernel and the quadric polynomial kernel, respectively.

### 4.3. Analysis of the Influencing Factors

#### 4.3.1. Factors Influencing Gas Permeability

The SVR models after parameter modification were used as the analytical models to regress the sample data and predict the gas permeability of the CMS membrane.

1. Regression results

**Figure 6:**
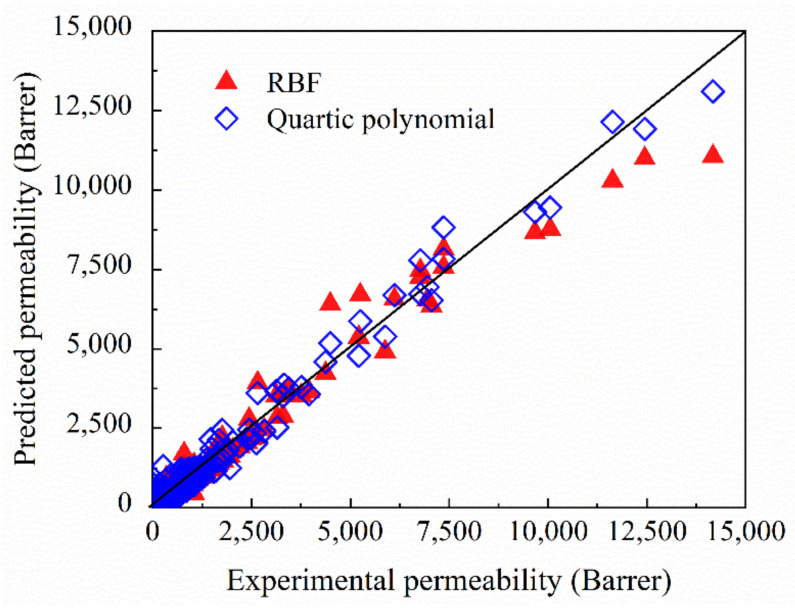
Comparison between predicted results and experimental data of the gas permeability by SVR method.

Figure 6 shows the comparison between the predicted results regressed by the SVR analytical models with two kernel functions and the experimental data of the gas permeability from all the precursors presented in Figure S1 [34,35,36,37,38,39,40,41,42,43]. The scatter points were evenly distributed on both sides of the diagonal, which indicated that the experimental data and the corresponding predicted values were close to each other.

Table 4 depicts the statistical indicators based on the regression results in Figure 6. Compared with the results in Table 2, the larger *R*^2^ and smaller *MAE* in Table 4 indicated that selecting the appropriate kernel function and optimizing the suitable parameters was important to improve the regression effect. The larger *RMSE*s in Table 4 was probably because a small number of data points were not predicted accurately in order to ensure the overall regression effect. The *R*^2^ of both models with optimized parameters were larger than 0.8, which revealed the high correlation between the results calculated by the SVR models after parameter adjustment and the experimental data. In addition, the regression effect of the SVR model with RBF kernel was better for the regression, due to the larger *R*^2^ and smaller *RMSE* and *MAE*.

2. Influencing factors analysis

**Figure 7:**
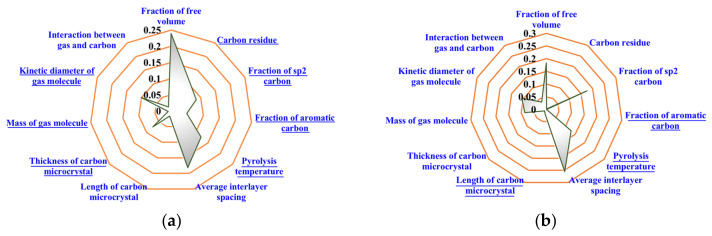
The normalized weights of the influencing factors on gas permeability regressed by SVR model with (**a**) RBF kernel; (**b**) quartic polynomial kernel (factors with underline are negative).

The radar map (Figure 7) was applied to represent the absolute values of the normalized weights for each influencing factor (calculated by |*w*_i_|/Σ|*w*_i_|, where *w_i_* is the original weight of the independent variable *i*). The factors with underline, which had a negative weight value, were negatively related to the permeability according to Equations (3) and (4), and were shown in absolute value in the radar map in order to realize an intuitive comparison among the weight of each independent variable. Moreover, the weights of the independent variables corresponding to the SVR models with the RBF kernel and the quartic polynomial kernel were different.

Figure 7a shows the regression result of the SVR model with the RBF kernel, which indicates that the main factors affecting the gas permeability of CMS membrane are the FFV of precursor, the average interlayer spacing, and the pyrolysis temperature. The precursor with the higher FFV would convert into the CMS membrane with a more developed microporous structure for gas transport and, therefore, the CMS membrane has higher gas permeability [44]. This is consistent with the experimental results in the previous work on the CMS membrane preparations from different precursors [36]. The average interlayer spacing could reflect the pore size of the CMS membrane to a certain extent. Wider interlayer spacing in the carbon matrix corresponded to a larger-sized micropore [36,37,38,42], which caused less hindrance to the gas molecules [50]. Higher final pyrolysis temperature would lead to a more thorough thermal condensation and a greater degree of shrinkage for the CMS membrane [9]. The SVR model with the RBF kernel could more reasonably determine the influences of each independent variable on gas permeability.

Figure 7b shows the regression by the model with the quartic polynomial kernel. The calculated results may be unreasonable because the weight of the factor “mass of gas molecule” was positive to the gas permeability. The gas permeability equals the product of gas adsorbability and gas diffusivity. The adsorbability has little relation to the molecule mass [5,8], while the diffusivity of gas molecule is inversely proportional to the square root of its molar mass in general [51], which indicates that the molar mass of gas should negatively relate to the gas permeability. Therefore, the SVR model with the quartic polynomial kernel may not suitable for the analysis of influencing factors on the gas separation performance of CMS membrane.

According to Figure 7a, showing the regression results by the SVR model with the RBF kernel, it could also be found that the weights of the gas properties were smaller than those of structural factors. Although the permeability of different gases in one CMS membrane varies greatly, the influences of membrane structure on the performance are stronger than those of gas properties regarding gas permeation. That is to say, the permeability of slow gas in the CMS membrane with a suitable structure would be higher than the one of fast gas in the membrane with a poor structure.

#### 4.3.2. Factors Influencing Gas Separation Performance

The SVR model with the RBF kernel, whose regression result of the gas permeability was more reasonable in Section 4.3.1, was selected to regress multiple factors to the characteristic distance (*d*) and resolve the weights in order to determine and analyze the key influential factors.

1. Regression results

**Figure 8:**
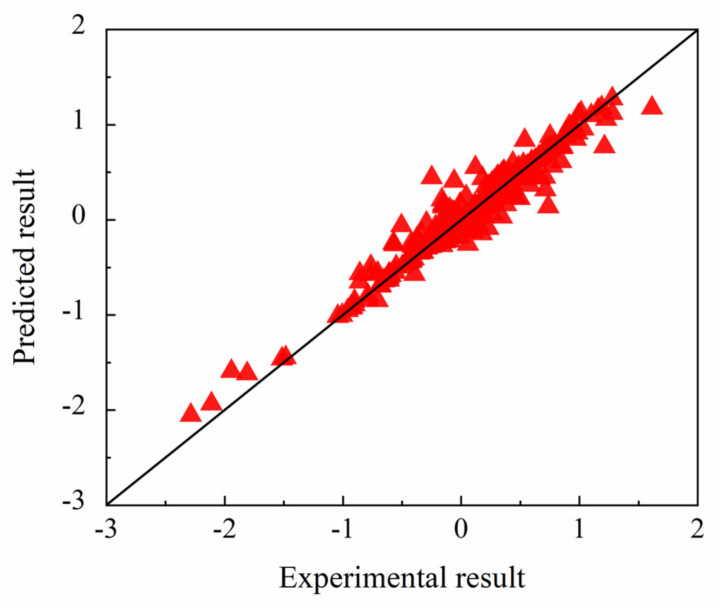
Comparison between predicted and experimental results of characteristic distance.

Figure 8 shows the comparison of the characteristic distance between the predicted results and the experimental data from all the precursors in Figure S1 [34,35,36,37,38,39,40,41,42,43]. The data points in Figure 8 were evenly distributed on the value domain and exhibited good representativeness, while the data points in Figure 6 were mostly concentrated on one side. The *R*^2^, *RMSE*, and *MAE* calculated from the predicted results and the experimental data were 0.932, 0.260, and 0.165, respectively, which indicated the reliability of the model.

2. Influencing factors analysis

**Figure 9:**
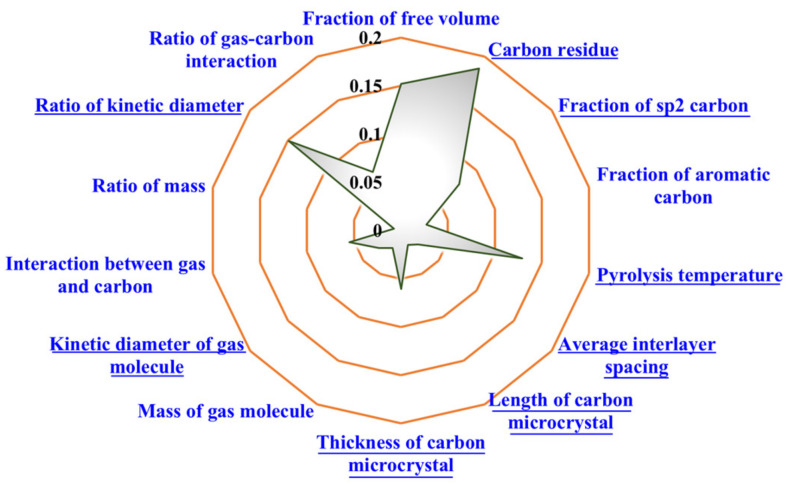
The normalized weight of the influencing factors on characteristic distance (factors with underline are negative).

Figure 9 shows the absolute values of the normalized weights. The most important factor influencing the synthetic gas permeation and separation performance is the porosity of the CMS membrane. The porosity is controlled by the carbon residue and the FFV of precursor, as well as the ratio between the kinetic diameters of the fast component and the slow component in the gas pair for separation. The smaller carbon residue of CMS membrane indicates that more small molecular groups would escape from the solid matrix during the thermal decomposition and more micropores would be created [42]. Such a structure is conducive to the transport of gas molecules in the CMS membrane, especially the fast gas [45,46]. Higher porosity corresponds to the larger FFV of the precursor, which is helpful to the gas permeation and separation of the CMS membrane [8,36]. The ratio of kinetic diameter, compared with other properties of gas molecules, is more influential, which manifests that the gap of effective diffusion between different gas molecules in the nanoscale micropores is the main reason for the selectivity of the CMS membrane. The analysis results according to Figure 9 could be helpful to the structural optimization and the separation performance improvement in the CMS membrane.

In addition, the weight of the average interlayer distance is relatively smaller than the one in Figure 7a. Increasing the interlayer distance, which would simultaneously increase the gas permeability of both fast gas and slow gas, may decline the selectivity of the gas pair which, thereby, has no significant effect on the gas separation performance of CMS membrane. The key influencing factors, i.e., the carbon residue and the FFV, are both related to the porosity of CMS membrane, as discussed above [44,45,46]. In order to improve the performance of gas permeation and selection comprehensively, priority should be given to increasing the porosity of materials of the CMS membrane if the structural parameters that need to be adjusted cannot reach the optimal level together. For the gas pairs with specific components, the interlayer spacing could be adjusted appropriately according to the kinetic diameter of gas molecules in order to improve the permeability.

## 5. Conclusions

The SVR method based on the machine learning technique was used to evaluate the relationship between the influencing factors and gas separation performance of self-manufactured CMS membrane. A simple index (*d*) based on the Robeson’s upper-bound line was put forward to quantitatively evaluate the gas separation performance of the CMS membrane, including both permeability and selectivity. Compared with the classical MLR method, the SVR method with the RBF kernel could acquire more accurate results. The main factors affecting the gas permeability of the CMS membrane are the FFV of the precursor, the average interlayer spacing of graphite-like carbon sheets, and the final carbonization temperature. The performance of gas permeability depends more on the structural characteristics than the properties of gas molecules. A reasonable structure design is the key to improve the gas permeability of the CMS membrane. The main factors affecting the gas separation performance of the CMS membrane are the carbon residue, the FFV of the precursor, and the ratio of gas kinetic diameters. If adjusting the structural parameters cannot reach the optimal performance, increasing the porosity of the CMS membrane could be preferentially considered by increasing the space for the transport of gas molecules.

## Figures and Tables

**Table 1:** The selected factors for analyzing the structural–performance relationship of CMS membrane.

Category	Contents
Precursor structure	Fractional free volume (FFV); carbon residue; fraction of sp2-hybrid carbon; fraction of carbon in aromatic rings
Carbonation condition	Pyrolysis temperature
Carbon microcrystal structure	Average interlayer spacing; length of carbon microcrystal; thickness of carbon microcrystal
Properties of permeated gas molecules	Mass; kinetic diameter; van der Waals potential between gas and carbon

**Table 2:** Statistical indicators of the results calculated by SVR models.

Kernel Function	*R* ^2^	*RMSE*	*MAE*
RBF	0.794	0.281	0.139
Polynomial	0.730	0.321	0.181
Linear	0.303	0.516	0.209
Sigmoid	–8.562	1.913	1.375

**Table 3:** Statistical indicators of the results calculated by MLR models.

Regression Method	*R* ^2^	*RMSE*	*MAE*
Linear	0.201	0.553	0.387
Ringe	0.204	0.552	0.386
Lasso	–0.060	0.637	0.409

**Table 4:** Statistical indicators of the results calculated by SVR analytical models.

Kernel Function	*R* ^2^	*RMSE*	*MAE*
RBF	0.841	0.413	0.129
Quartic polynomial	0.809	0.419	0.156

## Data Availability

Not applicable.

## Supplementary Materials

The following supporting information can be downloaded at: https://www.mdpi.com/article/10.3390/membranes12010100/s1, Figure S1: Chemical structures of precursors, Figure S2: Calculation of fractional free volume (FFV),Figure S3: Calculation of carbon structural parameters, Figure S4: Calculation of gas-carbon interaction, Tables S2 and S5: Parameters of gas pairs in the Robeson upper bound, Figure S6: The kernel functions used in the support vector regression, Figures S3 and S7: Variation of the statistical coefficients with the parameters of the kernel function.
