# Supplementary material for: Analysis of Influencing Factors on the Gas Separation Performance of Carbon Molecular Sieve Membrane Using Machine Learning Technique
Source: Membranes (Basel). 2022 Jan 17;12(1):100. doi: 10.3390/membranes12010100 (PMC8778672; doi:10.3390/membranes12010100)
Supplement: Supplementary file 1 [file membranes-12-00100-s001.zip › membranes-1554088-supplementary.pdf]

membranes

Article

Analysis of Influencing Factors on the Gas Separation Performance of Carbon Molecular Sieve Membrane using Machine Learning Technique

Yanqiu Pan [1], Liu He [1,2], Yisu Ren [3], Wei Wang [1,*] and Tonghua Wang [1]

[1] School of Chemical Engineering, Dalian University of Technology, Dalian 116024, China; yqpan@dlut.edu.cn (Y.P.); liuhe1992@hotmail.com (L.H.); wangth@dlut.edu.cn (T.W.)
[2] Jihua Laboratory, Foshan 528000, China
[3] Faculty of Science, The University of Melbourne, Melbourne 3010, Australia; szrenyisu@hotmail.com (Y.R.)
* Correspondence: dwwang@dlut.edu.cn (W.W.)

S.1 Precursors for the preparation of carbon molecular sieve (CMS) membrane

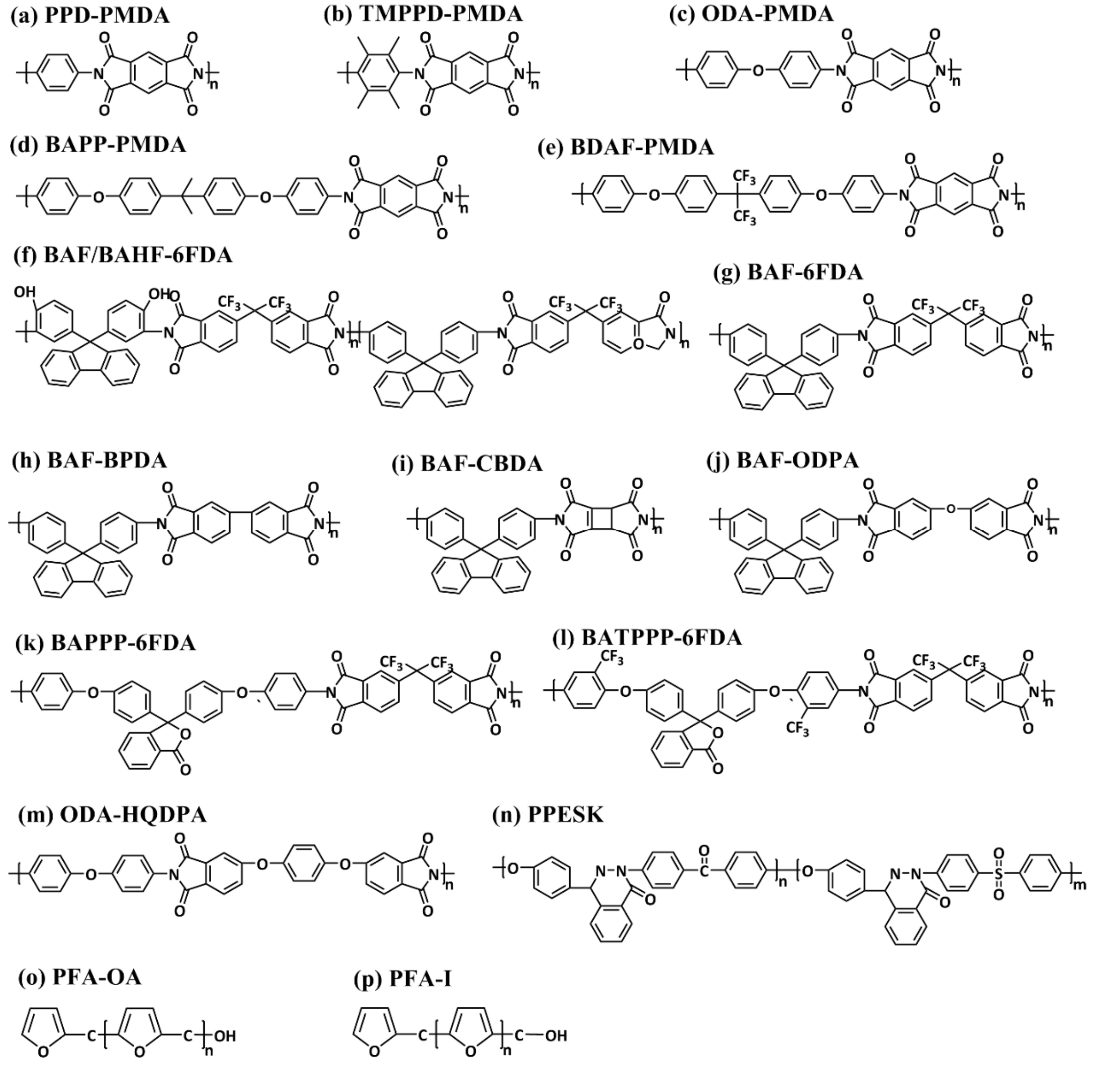

Figure S1. Chemical structures of precursors [1-10].

S.2 Calculation of fractional free volume (FFV)

The group contribution method was applied to the calculation of the FFV of the precursor structure as Equation S1[11]. The contribution of small functional groups in the polymeric chains to the volume of the polymer (V) and the volume occupied by the polymer chains (V_0) was calculated by Equations S2 and S3 respectively.

$$\mathrm{FFV} = \frac{V - V_0}{V} \quad \text{(S1)}$$

$$V_0 = 1.3 \sum_{k=1}^{K} (V_{\mathrm{W}})_k \quad \text{(S2)}$$

$$V = \sum_{k=1}^{K} \beta_k (V_{\mathrm{W}})_k \quad \text{(S3)}$$

where $(V_{\mathrm{W}})_k$ and β_k are the van der Waals volume and the pre-exponential factor of the group k, respectively.

S.3 Calculation of carbon structural parameters

The carbon microstructural parameters were determined from the X-ray diffraction (XRD) spectrum (Figure S1). The average interlayer spacing (d_{002}) of carbon was calculated by the Bragg equation (Equation S4) [12,13]:

$$\lambda = 2d_{002}\sin\theta_{002} \tag{S4}$$

where λ is the wavelength of X-ray, and θ_{002} is the diffraction angle of the 002 peak.

The L_c and L_a, which are respectively the sizes of 002 and 100 surface and correspond to the width and length of the carbon microcrystal in the membrane, were calculated by the Scherrer equation (Equations S5 and S6) [14-16]:

$$L_c = 0.94 \times \lambda / \beta_{002}\cos\theta_{002} \tag{S5}$$

$$L_a = 1.84 \times \lambda / \beta_{100}\cos\theta_{100} \tag{S6}$$

where β is half width of the corresponding peak.

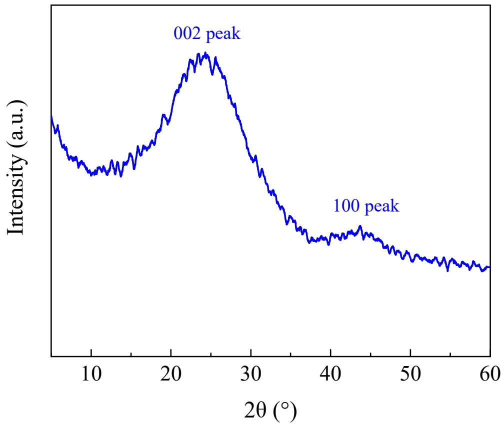

Figure S2. An example of XRD pattern of CMS membrane. The CMS membrane is derived from ODA-PMDA polyimide and the pattern is independently measured by the D/Max 2400 X-ray diffractometer produced by Rigaku Corporation.

S.4 Calculation of gas-carbon interaction

The gas-carbon interaction potential in this work was generated by van der Waals force, including dispersion force, orientation force and induced force [17-19].

Dispersion force is the interaction between gas molecules and carbon matrix due to instantaneous dipole. The potential energy E_L produced by the dispersion force is calculated by Equation S7:

$$E_{\mathrm{L}} = -\frac{3}{2} \cdot \frac{T_{\mathrm{c}} T_{\mathrm{g}}}{r^6} \cdot \frac{I_{\mathrm{c}} I_{\mathrm{g}}}{I_{\mathrm{c}} + I_{\mathrm{g}}} \tag{S7}$$

where r is the distance between carbon matrix and gas molecules, T_c and T_g are the polarizabilities of carbon and gas molecules, and I_c and I_g are the ionization potentials of carbon and gas molecules, respectively.

Orientation force, also known as electrostatic force, is the interaction between the gas molecules with permanent dipole moment or quadrupole moment and the polar groups on the surface of carbon matrix, which are respectively recorded as E_{k1} and E_{k2} and calculated by Equations S8 and S9.

$$E_{k1} = -\frac{2}{3} \cdot \frac{\mu_g^2 \mu_c^2}{r^6 T} \tag{S8}$$

$$E_{k2} = -\frac{\mu_c^2 Q_g^2}{r^8 kT} - \frac{7}{40} \cdot \frac{Q_c^2 Q_g^2}{r^{10} kT} \tag{S9}$$

where μ_c and μ_g are the intrinsic dipole moments of carbon and gas molecules respectively, and Q_c and Q_g are the corresponding quadrupole moments. T is the absolute temperature and k_B is Boltzmann constant.

Induced force is the interaction between the intrinsic polar groups on the surface of the carbon matrix and the gas molecules induced by the dipole and quadrupole moments from these groups. The potential energies from the forces induced by the dipole and quadrupole moments are respectively recorded as E_{D1} and E_{D2} and are calculated by Equation S10 and S11.:

$$E_{\mathrm{D1}} = -\frac{T_c\mu_g^2 + T_g\mu_c^2}{r^6} \tag{S10}$$

$$E_{\mathrm{D2}} = -\frac{3}{2}\frac{T_cQ_g^2 + T_gQ_c^2}{r^8} \tag{S11}$$

The potential energy from van der Waals force (E_v), which is the sum of the five interaction potential energies (Equation S12), were listed in Table 1:

$$E_{\mathrm{v}}(r) = E_{k1} + E_{k2} + E_{\mathrm{D1}} + E_{\mathrm{D2}} + E_{\mathrm{L}} \tag{S12}$$

Table S1. Estimate values of potential energies generated by van der Waals forces between gas molecule and solid carbon (kJ·mol^{-1}) [17-19]

Gas	E_L	E_{k1}	E_{k2}	E_{D1}	E_{D2}	E_V
CO_2	49.036	0	-66.00×10^{-23}	-2.45×10^{-8}	-1.734	-50.770
CH_4	29.244	0	-3.00×10^{-23}	-2.39×10^{-8}	-0.036	-29.280
O_2	18.371	0	-0.29×10^{-23}	-1.56×10^{-8}	-0.046	-18.417
N_2	21.294	0	-8.04×10^{-23}	-1.62×10^{-8}	-0.334	-21.628
H_2	-9.859	0	-1.56×20^{-23}	-1.12×10^{-8}	-0.034	-9.862

S.5 The parameters of the Robeson upper bound lines

Table S2. Parameters of gas pairs in the 2008 Robeson upper bound[20]

Gas pair	*k* (Barrer)	*n*
O_2/N_2	1,396,000	-5.666
CO_2/CH_4	5,369,140	-2.636
H_2/N_2	97,650	-1.484
H_2/CH_4	27,200	-1.107
CO_2/N_2	30,967,000	-2.888
N_2/CH_4	2,570	-4.507
H_2/CO_2	4,515	-2.302

S.6 The kernel functions used in the support vector regression

(1) Linear function:

$$\kappa(\boldsymbol{x}_i, \boldsymbol{x}_j) = \boldsymbol{x}_i^{\mathrm{T}} \boldsymbol{x}_j \tag{S13}$$

(2) Polynomial function (d is the degree of polynomial):

$$\kappa(\boldsymbol{x}_i, \boldsymbol{x}_j) = \left(\boldsymbol{x}_i^{\mathrm{T}} \boldsymbol{x}_j\right)^d, d \geq 1 \tag{S14}$$

(3) Radial basis function (RBF) (σ is called the bandwidth):

$$\kappa(\boldsymbol{x}_i, \boldsymbol{x}_j) = \exp\left(-\frac{\left\|\boldsymbol{x}_i - \boldsymbol{x}_j\right\|^2}{2\sigma^2}\right), \sigma > 0 \tag{S15}$$

(4) Sigmoid function (β_S and θ_S are the kernel parameters):

$$\kappa(\boldsymbol{x}_i, \boldsymbol{x}_j) = \tanh\left(\beta_S \boldsymbol{x}_i^{\mathrm{T}} \boldsymbol{x}_j + \theta_S\right), \beta > 0, \theta > 0 \tag{S16}$$

S.7 Parameter optimization of kernel functions

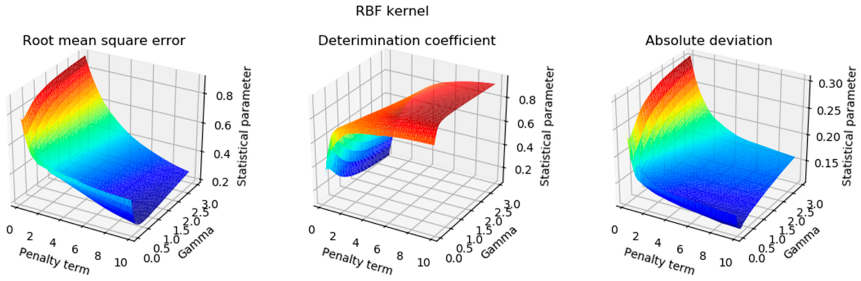

(a)

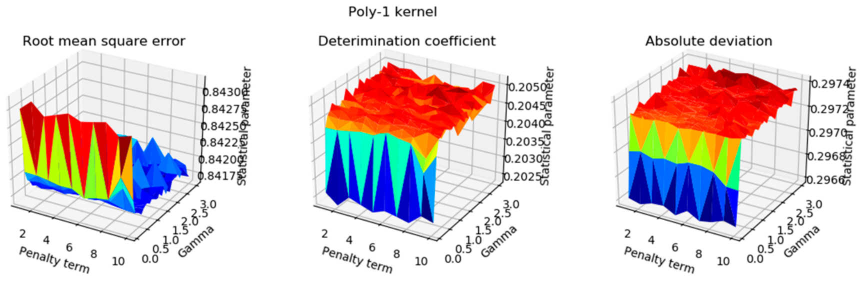

(b)

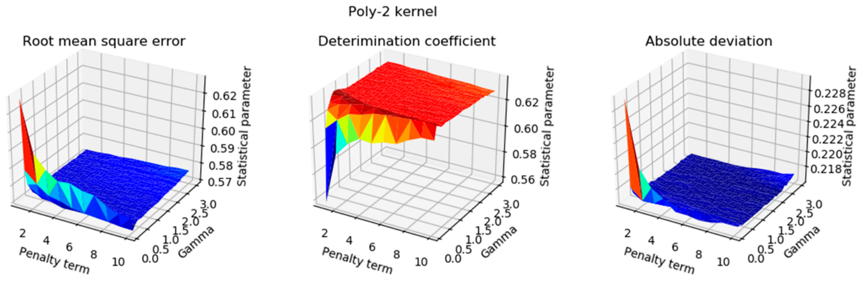

(c)

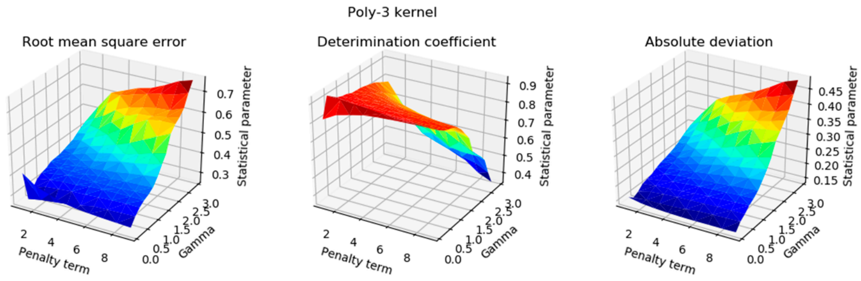

(d)

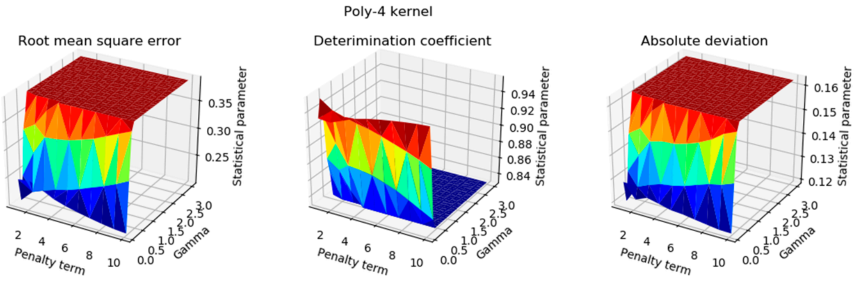

(e)

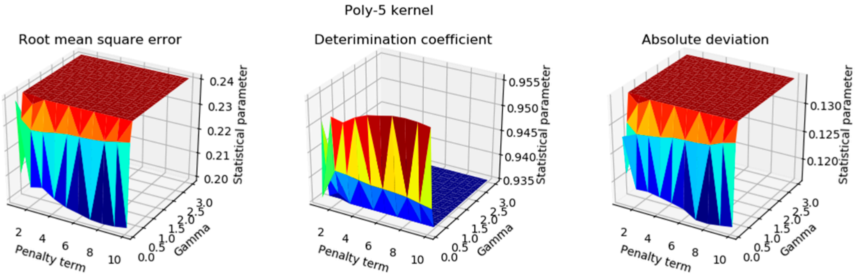

(f)

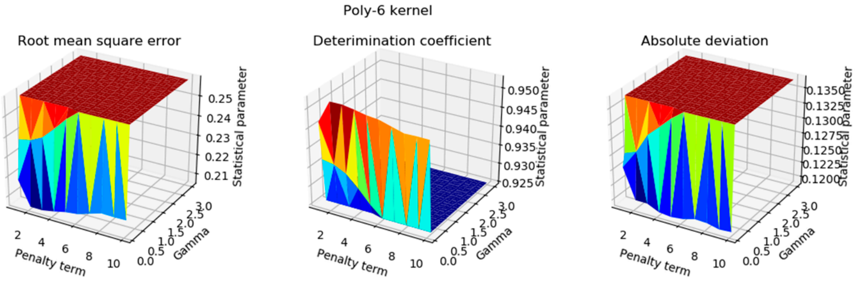

(g)

Figure S3. Variation of the statistical coefficients with the parameters of the kernel function. (a) corresponds to the RBF kernel; (b)~(g) correspond to the polynomial kernel.

References

1. Chen, A. The CO_2 Adsorption and Diffusion Performances in Carbon Membrane. M. Phil. Thesis, Dalian University of Technology, Dalian, 2012.
2. Qi, W. Effects of precursor chemical structure on the microstructure and gas permeability of carbon membrane. M. Phil. Thesis, Dalian University of Technology, Dalian, 2011.
3. Song, J. Carbon Molecular Sieve Membranes Derived from Polyimides for Gas Separation. M. Phil. Thesis, Dalian University of Technology, Dalian, 2017.
4. Xu, J. Effects of the Precursor Molecular Weight and ZSM-5 Addition on the Microstructure and Gas Permeability of Carbon Membrane. M. Phil. Thesis, Dalian University of Technology, Dalian, 2013.
5. Li, L. Preparation, Pyrolysis Mechanism and Structure Modification of Polyimide Based Carbon Membrane. Ph. D. Thesis, Dalian Univvversity of Technology, Dalian, 2013.
6. Hu, L. The Studies of Polyetherimide-based Pyrolytic Membrane by Low-temperature and Carbon Membrane. M. Phil. Thesis, Dalian University of Technology, Dalian, 2007.
7. Liu, S. Preoxidation Mechanism of Poly (phthalazione ether sulfone ketone)s and Preparation Technology Optimization of Their Carbon Membranes. Ph. D. Thesis, Dalian University of Technology, Dalian, 2008.
8. Pang, J. Effect of Different Solvents and Drying Methods on Structure and Gas Properties of Carbon Membranes. M. Phil. Thesis, Dalian University of Technology, Dalian, 2010.
9. Zhang, B. Preparation, Microstructure and Gas Separation Performance of Molecular Sieving Carbon Membranes. Ph. D. Thesis, Dalian University of Technology, Dalian, 2007.
10. Wang, X. The Preparation and Structure Properties of Carbon Membranes derived from Poly(furfuryl alcohol) for Gas Separation. M. Phil. Thesis, Dalian University of Technology, Dalian, 2007.
11. Park, J.Y.; Paul, D.R. Correlation and prediction of gas permeability in glassy polymer membrane materials via a modified free volume based group contribution method. *J Membrane Sci* **1997**, *125*, 23-39.
12. Cullity, B.D.; Stock, S.R. *Elements of X-Ray Diffraction* 3ed.; Pearson: New York, 2001.
13. Short, M.A.; Walker, P.L. Measurement of interlayer spacings and crystal sizes in turbostratic carbons. *Carbon* **1963**, *1*, 3-9.
14. Braun, A.; Bartsch, M.; Schnyder, B.; Kotz, R.; Haas, O.; Haubold, H.G.; Goerigk, G. X-ray scattering and adsorption studies of thermally oxidized glassy carbon. *J. Non-Cryst. Solids* **1999**, *260*.
15. Gurudatt, K.; Tripathi, V.S. Studies on changes in morphology during carbonization and activation of pretreated viscose rayon fabrics. *Carbon* **1998**, *36*, 1371-1377.
16. Wang, S.B.; Lu, G.Q. Effects of acidic treatments on the pore and surface properties of Ni catalyst supported on activated carbon. *Carbon* **1998**, *36*, 283-292.
17. Adamson, A.W. *Physical Chemistry of Surfaces*, 6 ed.; John Wiley & Sons: New Jersey, 1990.
18. Prausnitz, J.M.; Lichtenthaler, R.N.; Azevedo, E.G.d. *Molecular Thermodynamics of Fluid-Phase Equilibria*, 3 ed.; Prentice Hall: New Jersey, 1998.
19. M.Laurendeau, N. *Statistical Thermodynamics: Fundamentals and Applications*; Cambridge University Press: Cambridge, 2005.

20. Robeson, L.M. The upper bound revisited. *J Membrane Sci* **2008**, *320*, 390-400.
